# Hand Reconstruction Using Anterolateral Thigh Free Flap by Terminal Perforator-to-Digital Artery Anastomosis: Retrospective Analysis

**DOI:** 10.1055/a-2161-7419

**Published:** 2024-02-07

**Authors:** Jin Soo Kim, Ho Hyung Lee, Sung Hoon Koh, Dong Chul Lee, Si Young Roh, Kyung Jin Lee

**Affiliations:** 1 Department of Plastic and Reconstructive Surgery, Gwangmyeong Sungae General Hospital, Gwangmyeong, Republic of Korea

**Keywords:** microsurgical free flap, perforator flaps, hand injuries, reconstructive surgical procedures

## Abstract

This study aimed to analyze cases of anterolateral thigh (ALT) free flap used for hand reconstruction with terminal perforator-to-digital artery anastomosis. Patients who underwent ALT free flap placement with terminal perforator-to-digital artery anastomosis for hand reconstruction between January 2011 and August 2021 were included. The number, length, and diameter of the perforators and veins, flap size, and operative time were investigated through a retrospective review of charts and photographs. The occurrences of arterial thrombosis, venous thrombosis, arterial spasm, and flap necrosis were analyzed. In total, 50 patients were included in this study. The mean diameter and length of the perforators were 0.68 mm and 3.25 cm, respectively, and the mean number of veins anastomosed was 1.88, with a mean diameter of 0.54 mm. Complications included four cases of arterial thrombosis, one case of venous thrombosis, seven cases of partial necrosis, and one case of total flap failure. Regression analysis showed that a longer perforator was associated with arterial thrombosis whereas larger flap size and number of anastomosed veins were associated with partial necrosis ( *p* < 0.05). The terminal perforator-to-digital artery anastomosis offers advantages in using compact free flaps with short pedicle lengths to cover small hand defects.

## Introduction

Depending on the size and location of the defect, there are various methods for hand reconstruction, such as secondary intention, skin grafting, local flaps, pedicle flaps, and free flaps. Among these techniques, the free flap, which requires microsurgical expertise, offers versatility and robustness. The choice of flap is mainly dictated by the nature of the defect and surgeon preference and can be controversial depending on the situation. 1 2

In general, when a defect is large, larger flaps and vessels are required whereas smaller flaps and vessels are required for smaller defects. When the defect is located at the digital level, the size of the defect is small, necessitating the harvesting of a smaller flap. Therefore, it is important to select a suitable recipient vessel at an appropriate level and harvest perforators of appropriate length and diameter. 3

**Fig. 1:**
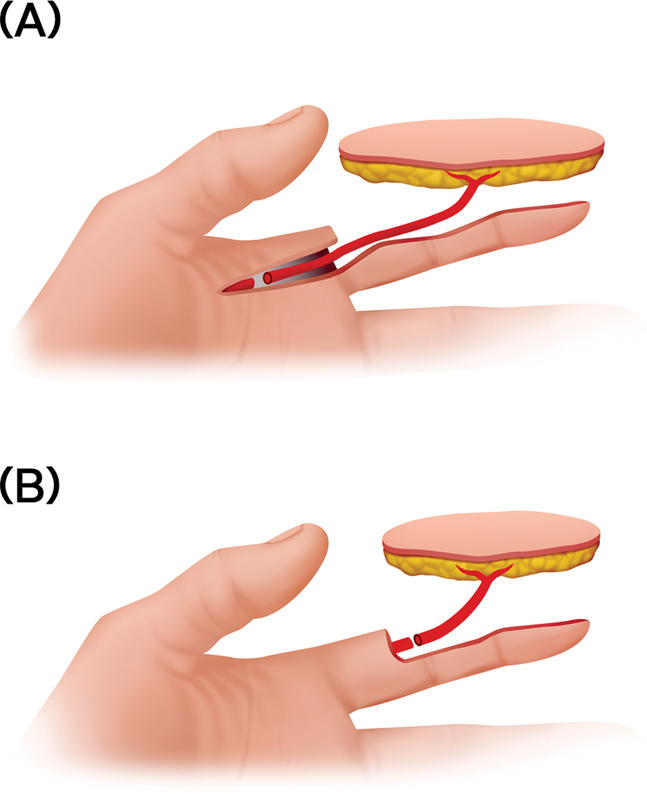
Schematic diagram of anastomosis. ( **A** ) Long perforator dissection up to the source vessel and recipient suitable artery. ( **B** ) Terminal perforator-to-digital artery anastomosis.

1. If a larger vessel, such as the metacarpal artery or wrist vessel, is used as the recipient vessel, a larger perforator is required, and dissection up to the source vessel is necessary ( Fig. 1A ).
2. In contrast, if the digital artery is used as the recipient vessel, a terminal perforator can be used without further dissection ( Fig. 1B ). This approach can save time; however, smaller diameter of the vessels increases technical difficulty.

The anterolateral thigh (ALT) free flap can be selected in both methods, providing versatility and pliability for hand reconstruction. It originates from the descending branch of the lateral circumflex femoral artery, enabling harvesting of perforators at selective anatomic levels. 4 The diameter of the perforator within the flap is comparable to the digital artery at the fascia level, and it increases as the length of the perforator extends toward the source vessel.

**Fig. 2:**
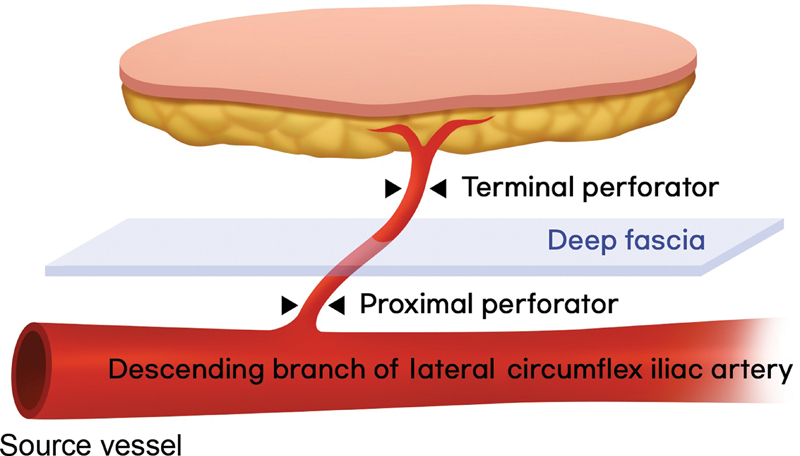
Using deep fascia as the criterion, we differentiated proximal and terminal perforators. This study included anterolateral thigh free flaps with terminal perforators harvested at the superficial level of the deep fascia.

From this perspective, this study included the perforator to the superficial level of the deep fascia as the terminal perforator ( Fig. 2 ) and used the digital artery as the recipient site for anastomosis ( Fig. 1B ). Through this, we conducted a prognosis analysis based on the division level of the perforator of a free flap intended to cover defects in the digit where skin perforators do not exist and aim to discuss the usefulness of the digital artery as the recipient artery.

## Ideas

This study was a retrospective chart review. We reviewed the medical records of patients who underwent ALT free flap coverage for digital reconstruction using terminal perforator-to-digital artery anastomosis. The course of the perforator was traced preoperatively using color Doppler imaging by the Department of Radiology, and the terminal perforator was confirmed by the surgeon by the hand Doppler during surgery.

Flap elevation was mainly performed in the superficial fascial plane due to the recipient site characteristics requiring thinner flaps. Additional defatting was performed both before and after the perforator division.

The pedicle division level of the perforator was more superficial than the deep fascia of the vastus lateralis muscle, denoting the terminal perforator. All perforators underwent end-to-end anastomosis with the digital artery. For the veins, vena comitans were end-to-end anastomosed with subcutaneous veins adjacent to the defect.

### Inclusion Criteria

Patients who met the following criteria were included: (1) those who underwent ALT free flap coverage for the upper extremity, (2) those who had the digital artery as the recipient, (3) those who had the terminal perforator end-to-end anastomosed to the recipient artery, (4) those who had follow-up data for more than 6 months, and (5) those who underwent surgery between January 2011 and December 2020.

### Exclusion Criteria

Patients who met any of the following criteria were excluded: (1) the location of the perforator division was closer to the source vessel than the deep fascia of the vastus lateralis muscle and (2) the recipient vessel used was proximal to the digital artery.

### Study Design

Charts and photographic data were reviewed retrospectively to investigate demographic data; operative time; flap size; pedicle diameter and length; the types, number, diameter, and length of the perforators and veins included in the study. The postoperative complications examined as outcomes included arterial thrombosis, venous thrombosis, arterial spasms, partial necrosis, and total flap failure.

### Statistical Analysis

We conducted a statistical analysis of the risk factors by performing regression tests with the independent variables of demographic data, pedicle profile, vein, etc., using the outcome as the dependent variable. Statistical analyses were performed using independent *t* -test and regression test in the Statistical Package for the Social Sciences Statistics for Windows (version 26.0; IBM Corp., Armonk, NY). We interpreted the results as statistically significant when the *p* -value was less than 0.05.

### Results

In total, 50 patients were included in this study. Among them, 44 (88%) were men, and the mean age was 47.46 years. The mean perforator diameter was 0.68 mm. The mean perforator length was 3.25 cm. The mean number of vein anastomoses performed was 1.88. The mean vein diameter was 0.54 mm. The mean flap size was 62.03 cm ^2^ . The mean operative time was 253.3 minutes. Complications included arterial thrombosis in four cases, venous thrombosis in one case, partial necrosis in seven cases, and total flap failure in one case ( Table 1 ).

**Table 1:** 

Demographic data ( *n* = 50)	
Men ( *n* , %)	44 (88%)
Age, years (mean, SD)	47.42 (± 13.5)
Flap size, cm ^2^ (mean, SD)	62.03 (± 36.77)
Operative time (min)	253.3 (± 92.08)
Vessel profiles	
Pedicle length, cm (mean, SD)	3.25 (± 1.02)
Pedicle diameter, mm (mean, SD)	0.68 (± 0.24)
Number of veins (mean, SD)	1.88 (± 0.56)
Vein diameter, mm (mean, SD)	0.54 (± 0.12)
Complications ( *n* , %)	
1. Arterial insufficiency	4 (8%)
2. Venous insufficiency	1 (2%)
3. Arterial spasm	0
4. Hematoma	0
5. Partial necrosis	7 (14%)
6. Total flap failure	1 (2%)

Abbreviation: SD, standard variation.

Values are presented as number (%).

Regression analysis was conducted using constant variables for each complication as independent variables. The results revealed that a longer pedicle was associated with a higher incidence of arterial thrombosis. Additionally, a lower number of anastomosed veins and larger flap size were associated with a higher occurrence of partial necrosis ( *p* < 0.05). However, no statistically significant correlation was found between other complications and variables ( Table 2 ). No statistically significant difference was observed in the occurrence of venous thrombosis or total flap failure, with one case each. In the case of venous thrombosis, a revision surgery was performed because of congestion, and circulation was restored after venorrhaphy, without any further complications.

**Table 2:** 

Logistic regression analysis of complications ( *n* = 50)		
	OR (95% CI)	*p* -Value a
1. Arterial thrombosis ( *n* = 4)		
Pedicle length (cm)	**9.65 (1.04–89.33)**	** 0.046 b**
Pedicle diameter (mm)	0.62 (<0.01–209.40)	0.873
Operative time (min)	1.00 (0.97–1.03)	0.906
Flap size (cm ^2^ )	0.99 (0.94–1.05)	0.761
Age (years)	1.07 (0.95–1.20)	0.259
2. Partial necrosis ( *n* = 7)		
Pedicle length (cm)	0.93 (0.26–3.28)	0.904
Pedicle diameter (mm)	<0.01 (<0.01–6.15)	0.153
Number of veins	**0.06 (<0.01–0.91)**	** 0.043 b**
Operative time (min)	1.01 (1.01–1.14)	0.240
Flap size (cm ^2^ )	**1.07 (1.01** – **1.14)**	** 0.023 b**
Age (years)	1.14 (0.99–1.31)	0.066

Abbreviations: CI, confidence interval; OR, odds ratio.

Note: Bold values reflect statistical significance.

a Logistic regression test.

b *p* -Value <0.05 indicates statistical significance.

## Discussion

**Fig. 3:**
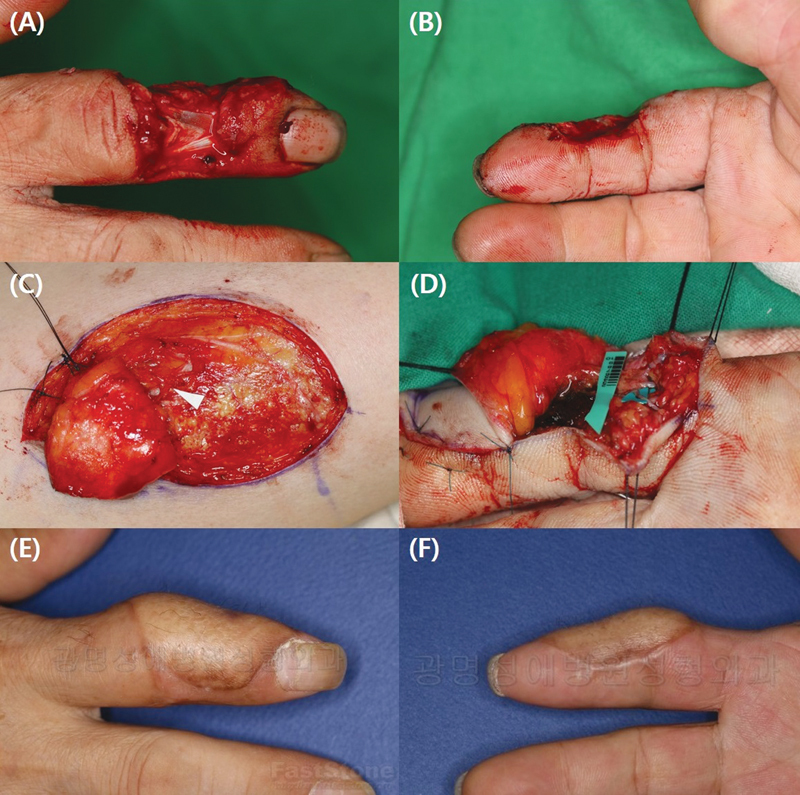
( **A** , **B** ) Patient with soft tissue defect of middle phalanx from the dorsal side to the voloradial side, which required a pliable flap coverage with versatility. ( **C** ) The anterolateral thigh free flap was harvested with the terminal perforator secured at the superficial fascial plane. ( **D** ) The perforator was anastomosed to the radial digital artery located at the proximal phalanx. ( **E** , **F** ) Follow-up photographs taken 3 months later.

Reconstruction options for severe defects at the digital level of the hand vary depending on the size of the defect. For smaller fingertip defects, a second toe pulp free flap, innervated radial artery superficial palmar branch perforator free flap, and hypothenar perforator free flap can be considered, including glabrous skin and sensation, but with limitations in harvest size. 5 6 For larger defects, the lateral arm free flap, ALT free flap, and other large flaps are feasible options. If these flaps' perforators are elevated to the source vessel level for use in smaller hand defects, there is a possibility of a vascular size mismatch with the recipient digital artery. Moreover, such discrepancies can lead to unpredictable results owing to variables, such as turbulence and shearing forces. 3 Therefore, when an ALT free flap is applied with the digital artery as the recipient, it is suitably sized for anastomosis with the terminal perforator ( Fig. 3 ).

One drawback is the difficulty associated with the anastomosis of small-diameter vessels. However, with the development of supermicrosurgery, manipulation of small vessels with diameter of 0.8 mm or less has become possible. 7 8 9 Recent studies have also shown that there is no significant difference in the success rates of anastomosing small arteries between fingertip and proximal replantation. 10 11 From this perspective, we studied free flap using small artery with terminal artery-to-digital artery anastomosis.

### Arterial Thrombosis

The results showed that arterial thrombosis was associated with a longer perforator; however, the arterial thrombosis diameter did not have a statistically significant impact. However, in other studies, the perforator length has not considered a risk factor for free flap thrombosis. 12 13 14 15 Three hypotheses were proposed based on these results. First, handling a long perforator may increase the risk of vascular injury. As vessels extend distally, they experience a decrease not only in diameter but also in the thickness of the vessel wall, which poses a challenge. Thus, the manipulation of long vessels could contribute to vascular injury and thrombosis. 16

Second, thrombosis occurs more frequently as surgical time increases, leading to longer exposure of the subendothelial structures to blood flow. 17 18 Using a shorter perforator when requiring the same vessel length for blood supply reduces recipient and perforator dissections, which shortens the surgical time. In other words, the terminal perforator-to-digital anastomosis may have a relatively protective effect against arterial thrombosis.

Third, arterial thrombosis was expected to occur more frequently as the diameter of the pedicle decreased; however, the difference was not statistically significant. In cases with differences in diameter, factors, such as turbulence, can occur within the vessel owing to the viscosity of blood, increasing the risk of thrombosis. 3 In this study, smaller vessels were suitably anastomosed, which may have reduced risk. In addition, we discuss this aspect in relation to spasms. It is important to emphasize that spasms did not occur in this study despite their small diameters. Although vasospasm seems less significant in thrombus formation, as spasm alone does not increase clot formation, 19 a narrow lumen results in decreased blood flow, and reducing the number of thrombocytes necessary to occlude the vessel can synergize with prothrombotic factors. 20

### Arterial Spasm

While arterial spasm did not occur in this study group, it is generally recognized that the incidence of spasm increases as the diameter of the vessel decreases. During the same period, 100 patients, including the existing study group, underwent ALT free flap upper extremity reconstruction using terminal perforators anastomosing various recipient arteries. Among them, spasms occurred in four individuals. Regression analysis results ( *n* = 100) showed a tendency for arterial spasms to occur as the diameter of the perforator decreased ( Table 3 ). This difference can be attributed to the distinct roles of recipient arteries. The digital artery plays a critical role in the main circulation of the digits and typically runs in two parallel pairs from the proximal part of the finger toward the fingertip, complementing each other. In contrast, other recipient arteries corresponding to the branches of the hand and forearm were less resilient to spasms. Therefore, the use of digital arteries as recipient vessels for digital defects has inherent advantages. 9 21

**Table 3:** 

Logistic regression analysis of complications ( *n* = 100)		
	OR (95% CI)	*p* -Value a
1. Arterial thrombosis ( *n* = 7)		
Pedicle length (cm)	**3.374 (1.29–8.85)**	**0.013**
Pedicle diameter (mm)	0.10 (<0.01–3.06)	0.184
Operative time (min)	1.01 (0.99–1.02)	0.386
Flap size (cm ^2^ )	1.00 (0.98–1.02)	0.906
Age (year)	1.05 (0.97–1.14)	0.198
2. Arterial spasm ( *n* = 4)		
Pedicle length (cm)	0.526 (0.162–1.708)	0.285
Pedicle diameter (mm)	**<0.01 (<0.01** – **0.497)**	**0.038**

Abbreviations: CI, confidence interval; OR, odds ratio.

Note: Bold values reflect statistical significance.

a Logistic regression test. *p* -Value <0.05 indicates statistical significance.

### Partial Necrosis

**Fig. 4:**
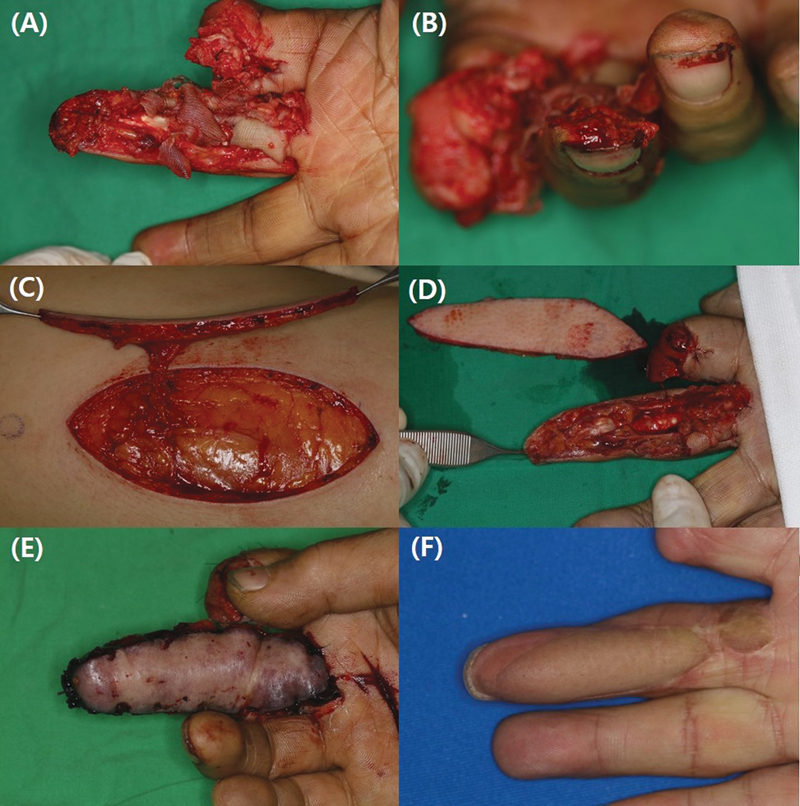
( **A** , **B** ) A volar side soft tissue defect occurred on the entirety of the right middle finger, requiring soft tissue coverage from the proximal phalanx to the fingertip. ( **C** , **D** ) An anterolateral thigh free flap was harvested to match the size of the defect and a terminal perforator was secured for anastomosis to the radial digital artery of the middle finger. ( **E** ) The following day, congestion occurred with venous thrombosis, which was resolved using venorrhaphy during a revisional surgery. ( **F** ) Follow-up photographs taken 1 year later.

Partial necrosis was associated with the number of veins and larger flap size. Anastomosing additional veins reduces congestion and reoperation rates. 17 22 Although the number of cases was insufficient for statistical analysis, flap congestion with venous thrombosis occurred in one case where only one vein was anastomosed; however, no further complications were observed after revision surgery ( Fig. 4 ). The distance from the perforator to the flap margin and flap size can increase the risk of partial necrosis. Minimizing the flap size within the perforasome governed by the perforator decreases the blood supply requirements, which can act as a protective factor against necrosis. 7 21 23

The limitations of this study included the following. It was a retrospective study, and a comparative analysis could not be conducted under identical clinical situations. The recipient was not standardized, and factors, such as contracture, crushing state, vascular integrity, diabetes, and obesity may have affected the outcome.

Additionally, the terminal perforator was positioned more superficially than the deep fascia; however, anatomical variations primarily occurred in the perforator of the descending branch of the lateral circumflex iliac artery. Consequently, the length and nature of the secured pedicle may vary, depending on whether it runs parallel to the deep fascia. Also, there were differences in the achievable length of the perforators based on whether the perforator coursed perpendicularly or obliquely within the adipose tissue layer. 24

Despite its limitations, this study confirmed that the terminal perforator-to-digital artery anastomosis approach in digit reconstruction cannot only reduce surgery time by reducing dissection, but also lower the risk of thrombosis, making the benefits outweigh the disadvantages of using smaller vessel diameters. Hence, when a digital defect occurs and a wider range is desired to be covered by a free flap rather than a glabrous skin flap, it could be argued that approaching from Fig. 1B rather than Fig. 1A and performing the division of the perforator only as much as necessary in a more superficial plane to anastomose with the digital artery could be beneficial.

**Fig. 5:**
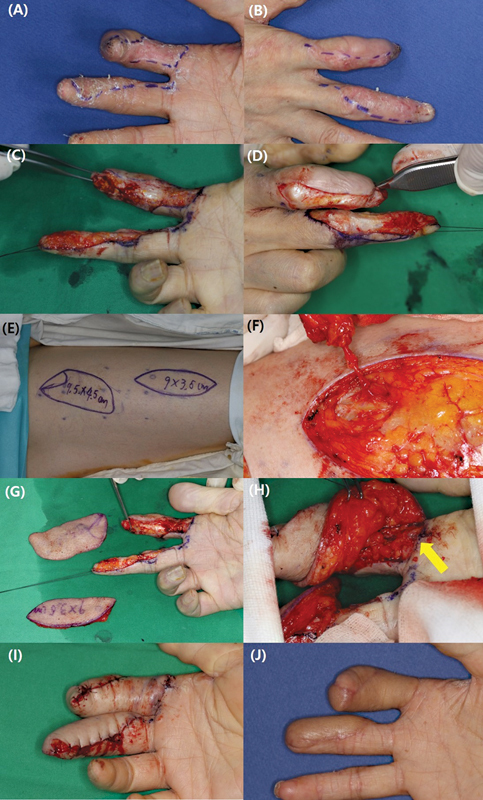
( **A** – **D** ) Two subtotal defects occurred in the index and middle fingers after carcinoma resection. ( **E** , **F** ) Two free flaps based on the terminal perforator were harvested from the anterolateral thigh to cover each defect. ( **G** , **H** ) Both flaps were applied to each finger, and the perforator was anastomosed to the digital artery (yellow arrow). ( **I** ) The flaps were successfully inset onto each finger. ( **J** ) Follow-up photographs taken 1 year later.

Additionally, considering a freestyle approach with perforators beyond the source vessel anatomy is worthwhile, as it builds upon the useful concept of applying a terminal perforator. 25 By breaking away from the traditional reliance on the descending branch of the lateral circumflex femoral artery, multiple thin, pliable flaps can be applied using terminal perforators from various source arteries and variants ( Fig. 5 ). This technique has the potential to cover multiple digit defects when faced with donor limitations. The current study on terminal perforator-to-digital artery anastomosis provides support as an extension of this concept.

Elevating the terminal perforator vessel and anastomosing it to the digital artery can be useful for reconstructing digital defects using an ALT free flap.

## References

[1] Holm A Zachariae L Fingertip lesions. An evaluation of conservative treatment versus free skin grafting Acta Orthop Scand 1974 45 03 382 392 4600692 10.3109/17453677408989160

[2] Kozin E D Sethi R K Herr M Comparison of perioperative outcomes between the supraclavicular artery island flap and fasciocutaneous free flap Otolaryngol Head Neck Surg 2016 154 01 66 72 26467355 10.1177/0194599815607345

[3] Doh G Kim B Lee D Hemodynamic principles in free tissue transfer: Vascular changes at the anastomosis site Arch Hand Microsurg 2021 26 285 292

[4] Valdatta L Tuinder S Buoro M Thione A Faga A Putz R Lateral circumflex femoral arterial system and perforators of the anterolateral thigh flap: an anatomic study Ann Plast Surg 2002 49 02 145 150 12187341 10.1097/00000637-200208000-00006

[5] Yang J W Kim J S Lee D C The radial artery superficial palmar branch flap: a modified free thenar flap with constant innervation J Reconstr Microsurg 2010 26 08 529 538 20648418 10.1055/s-0030-1262953

[6] Lee D C Kim J S Ki S H Roh S Y Yang J W Chung K C Partial second toe pulp free flap for fingertip reconstruction Plast Reconstr Surg 2008 121 03 899 907 18317138 10.1097/01.prs.0000299945.03655.0d

[7] Min K Hong J P Suh H P Risk factors for partial flap loss in a free flap: a 12-year retrospective study of anterolateral thigh free flaps in 303 lower extremity cases Plast Reconstr Surg 2022 150 05 1071e 1081e 10.1097/PRS.0000000000009646 36067478

[8] Velazquez-Mujica J Losco L Aksoyler D Chen H C Perforator-to-perforator anastomosis as a salvage procedure during harvest of a perforator flap Arch Plast Surg 2021 48 04 467 469 34352962 10.5999/aps.2020.02194 PMC8342250

[9] Diaz-Abele J Hayakawa T Buchel E Anastomosis to the common and proper digital vessels in free flap soft tissue reconstruction of the hand Microsurgery 2018 38 01 21 25 27392815 10.1002/micr.30066

[10] Hong M K Park J Koh S H Analysis of outcomes of tamai zone I digital replantation in cases of severe crushing injury Arch Hand Microsurg 2020 25 297 303

[11] Hattori Y Doi K Sakamoto S Yamasaki H Wahegaonkar A Addosooki A Fingertip replantation J Hand Surg Am 2007 32 04 548 555 17398367 10.1016/j.jhsa.2007.01.019

[12] Masoomi H Clark E G Paydar K Z Predictive risk factors of free flap thrombosis in breast reconstruction surgery Microsurgery 2014 34 08 589 594 24665051 10.1002/micr.22250

[13] Lese I Biedermann R Constantinescu M Grobbelaar A O Olariu R Predicting risk factors that lead to free flap failure and vascular compromise: a single unit experience with 565 free tissue transfers J Plast Reconstr Aesthet Surg 2021 74 03 512 522 33039304 10.1016/j.bjps.2020.08.126

[14] Biben J A Atmodiwirjo P Free flap thrombosis in patients with hypercoagulability: a systematic review Arch Plast Surg 2019 46 06 572 579 31775211 10.5999/aps.2019.00738 PMC6882692

[15] Chang E I Zhang H Liu J Yu P Skoracki R J Hanasono M M Analysis of risk factors for flap loss and salvage in free flap head and neck reconstruction Head Neck 2016 38 01 E771 E775 25914303 10.1002/hed.24097

[16] Venkatramani H Sabapathy S R Fingertip replantation: technical considerations and outcome analysis of 24 consecutive fingertip replantations Indian J Plast Surg 2011 44 02 237 245 22022034 10.4103/0970-0358.85345 PMC3193636

[17] Suyama Y Yagi S Fukuoka K Risk factors of free flap complications in reconstruction for head and neck cancer Yonago Acta Med 2022 65 03 215 225 36061574 10.33160/yam.2022.08.007 PMC9419227

[18] Acland R Thrombus formation in microvascular surgery: an experimental study of the effects of surgical trauma Surgery 1973 73 05 766 771 4697094

[19] Bentz M L Sheppeck R A Macpherson T Vasospasm and platelet deposition in human arteries: effects of topical methylene blue Plast Reconstr Surg 1991 88 05 851 859 1924572 10.1097/00006534-199111000-00018

[20] Johnson P C Barker J H Thrombosis and antithrombotic therapy in microvascular surgery Clin Plast Surg 1992 19 04 799 807 1285048

[21] Morris S F Tang M Almutari K Geddes C Yang D The anatomic basis of perforator flaps Clin Plast Surg 2010 37 04 553 570, xixi. 20816512 10.1016/j.cps.2010.06.006

[22] Matthews J LK Alolabi N Farrokhyar F Voineskos S H One versus 2 venous anastomoses in free flap surgery: a systematic review and meta-analysis Plast Surg (Oakv) 2018 26 02 91 98 29845046 10.1177/2292550317740693 PMC5967168

[23] Saint-Cyr M Wong C Schaverien M Mojallal A Rohrich R J The perforasome theory: vascular anatomy and clinical implications Plast Reconstr Surg 2009 124 05 1529 1544 20009839 10.1097/PRS.0b013e3181b98a6c

[24] Suondoh M S Sulaiman W AW Halim A S Distribution of anterolateral thigh flap perforator vessels and its clinical applications in Malaysian population Arch Hand Microsurg 2020 25 219 224

[25] Wei F C Mardini S Free-style free flaps Plast Reconstr Surg 2004 114 04 910 916 15468398 10.1097/01.prs.0000133171.65075.81

